# Costs and health-related quality of life in Alpha-1-Antitrypsin Deficient COPD patients

**DOI:** 10.1186/s12931-017-0543-8

**Published:** 2017-04-17

**Authors:** Florian M. Karl, Rolf Holle, Robert Bals, Timm Greulich, Rudolf A. Jörres, Annika Karch, Armin Koch, Stefan Karrasch, Reiner Leidl, Holger Schulz, Claus Vogelmeier, Margarethe E. Wacker, for the COSYCONET Study Group

**Affiliations:** 1 Institute of Health Economics and Health Care Management, Helmholtz Zentrum München, GmbH, German Research Center for Environmental Health, Comprehensive Pneumology Center Munich (CPC-M), Member of the German Center for Lung Research, Ingolstaedter Landstr. 1, 85764 Neuherberg, Germany; 2 Institute for Medical Information Processing Biometrics and Epidemiology (IBE) Ludwig-Maximilians-Universität München (LMU), Marchioninistr. 15, 81377 Munich, Germany; 3 Department of Internal Medicine V – Pulmonology, Allergology, Respiratory Intensive Care Medicine, Saarland University Hospital, Kirrberger Straße 1, 66424 Homburg, Germany; 4 Department of Respiratory Medicine, University of Marburg, University Giessen and Marburg Lung Center (UGMLC), Member of the German Center for Lung Research, Baldingerstraße, 35043 Marburg, Germany; 5 Institute and Outpatient Clinic for Occupational, Social and Environmental Medicine, Ludwig-Maximilians-Universität München, Ziemssenstr. 1, 80336 Munich, Germany; 6 Institute for Biostatistics, Hannover Medical School, Carl-Neuberg-Str. 1, 30625 Hannover, Germany; 7 Institute of Epidemiology I, Helmholtz Zentrum München, GmbH, German Research Center for Environmental Health, Comprehensive Pneumology Center Munich (CPC-M), Member of the German Center for Lung Research, Ingolstaedter Landstr. 1, 85764 Neuherberg, Germany

**Keywords:** COPD, Alpha-1-Antitrypsin Deficiency, Direct costs, Indirect costs, Health-related quality of life, Augmentation therapy

## Abstract

**Background:**

Alpha-1-Antitrypsin Deficiency (AATD) is an economically unexplored genetic disease.

**Methods:**

Direct and indirect costs (based on self-reported information on healthcare utilization) and health-related quality of life (HRQL, as assessed by SGRQ, CAT, and EQ-5D-3 L) were compared between 131 AATD patients (106 with, 25 without augmentation therapy (AT)) and 2,049 COPD patients without AATD participating in the COSYCONET COPD cohort. The medication costs of AT were excluded from all analyses to reveal differences associated with morbidity profiles. The association of AATD (with/without AT) with costs or HRQL was examined using generalized linear regression modelling (GLM) adjusting for age, sex, GOLD grade, BMI, smoking status, education and comorbidities.

**Results:**

Adjusted mean direct annual costs were €6,099 in AATD patients without AT, €7,117 in AATD patients with AT (excluding costs for AT), and €7,460 in COPD patients without AATD. AATD with AT was significantly associated with higher outpatient (+273%) but lower inpatient (−35%) and medication costs (−10%, disregarding AT) compared with COPD patients without AATD. There were no significant differences between groups regarding indirect costs and HRQL.

**Conclusion:**

Apart from AT costs, AATD patients tended to have lower, though not significant, overall costs and similar HRQL compared to COPD patients without AATD. AT was not associated with lower costs or higher HRQL.

**Trial registration:**

NCT01245933

**Electronic supplementary material:**

The online version of this article (doi:10.1186/s12931-017-0543-8) contains supplementary material, which is available to authorized users.

## Background

Alpha-1-antitrypsin (AAT) is an anti-protease that protects tissues – mainly lung parenchyma – from damage by enzymes released from inflammatory cells. Thus a deficiency of AAT can lead to uninhibited lung parenchymal destruction and the development of chronic obstructive pulmonary disease (COPD), particularly lung emphysema [1]. There are about 100 different genetic mutations leading to dysfunction of AAT or Alpha-1-antitrypsin deficiency (AATD) [2] which is also associated with liver cirrhosis [3] and overexpression of pro-apoptotic pathways [4].

AATD is a very rare but also highly underdiagnosed disease [5, 6]. The prevalence of deficiency related alleles (PI*ZZ and PI*SZ) in the world population is estimated to be 0.1% for the PI*ZZ and 0.7% for the PI*SZ genotype [7]. However, assessment of the true prevalence is difficult, as there are no population-based, large-scale screening studies [1], due to the difficulties of conducting population-based studies, poor awareness of the disease, perceived lack of treatment efficacy, and complexity of testing algorithms [8, 9].

COPD patients with underlying AATD differ from COPD patients without this deficiency e.g. with regard to smoking history, comorbidities and exercise-related desaturation in arterial blood [10, 11]. However, standard COPD therapy is recommended for AATD patients [12]. Comorbidities, which have been identified as a major driver of excess costs as well as of loss in health-realted quality of life (HRQL) in COPD patients [13, 14], may differ between patients with and without AATD.

Therefore, the aim of this cross-sectional analysis was to compare the annual direct and indirect costs as well as generic and disease-specific HRQL between COPD patients with and without AATD. To our knowledge, this has not yet been done before in a large-scale study. To identify costs related to (co-)morbidity profiles in AATD compared to non-AATD patients, the tremendous cost bias from alpha-1-proteinase inhibitor augmentation therapy (AT) was circumvented by excluding these costs from the analysis. As there is an ongoing controversy about the efficacy of AT therapy [1, 15], we also examined differences regarding (non-AT) direct and indirect costs and HRQL between augmented and non-augmented AATD patients.

## Methods

### Data and study design

**Fig. 1:**
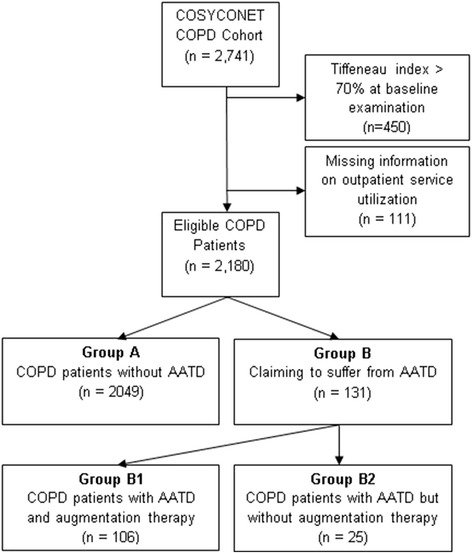
Patient flow diagram

Data was obtained from the baseline examination of the German multicentre COPD cohort COSYCONET (*German COPD and Systemic Consequences – Comorbidities Network*). The study included 2,741 subjects with a clinical diagnosis of COPD. Recruitment took place in 31 study centres between September 2010 and December 2013. Participants had to be ≥40 years of age and stable. Detailed information on recruitment and exclusion criteria can be found in Karch et al. [16]. COPD was defined according to the GOLD criteria after performing standardized post-bronchodilator spirometry [17]. For GOLD staging, reference values according to the Global Lung Initiative (GLI) were used [18]. 450 subjects with a Tiffeneau index above 70% at baseline examination were excluded from this analysis (Fig. 1).

AATD patients were identified by using self-reports on AATD and its genotype, AT from the medication list and the measured AAT serum levels. Participants with the heterozygous genotype PiMZ were classified as COPD patients without AATD. If a person answered that they did not know his or her genotype and was not receiving AT, an AAT serum level cut-off-point of <50 mg/dl was selected to distinguish COPD patients with and without AATD. With *n* = 131 patients (6.0%), including 106 patients with AT and 25 without AT, AATD was overrepresented in COSYCONET (Fig. 1).

### Healthcare utilisation, work absenteeism and calculation of costs

Healthcare utilisation was assessed using standardised questionnaires and interviews. The calculation of costs has been described previously [14]. In brief, outpatient physician visits (last 3 months, subdivided by 13 specialists), and physiotherapist visits, rehabilitation and days spent in hospital (past 12 months) were considered for the calculation of direct healthcare costs. These quantities were multiplied by the German unit costs (Additional file 1) of the year 2012 [19]. Furthermore, currently used prescribed pharmaceuticals were considered. Utilization of pharmaceuticals was estimated based on patients’ information on the drug code, defined daily doses [20] and German pharmacy retail prices of the year 2012 [21]. For the calculation of costs per year all costs were extrapolated to 12 months. In case of augmented AATD patients, the estimated mean annual costs of AT, €72,255 [21], were subtracted from the medication costs. Indirect costs as a consequence of production losses [22] included the temporary inability to work and premature retirement for participants aged < 65 years. For cost calculations the human capital approach was applied that accounts full labour costs for all sick days (€177 per missed day in 2012) [22]. Premature retirement (<65 years) was valued with mean annual labour costs of €37,126 per year [23]. As there is an ongoing discussion about the appropriate valuation of indirect costs [22], the friction cost approach was additionally applied. This means, that missed working days were calculated at only €142 and only for the first 50 days of work absenteeism, and costs for premature retirement were omitted [24].

### Health-related quality of life

Participants completed the Saint-George’s-Respiratory-Questionnaire (SGRQ) [25] and the COPD Assessment Test (CAT) [26]. The scoring of the SGRQ ranges from 0 to 100 whereas CAT ranges from 0 to 40 with higher scores indicating worse HRQL [25, 26]. The generic EuroQol 5 dimensions (EQ-5D-3 L) questionnaire including Visual Analogue Scale (VAS) was used in addition as an established German EQ-5D-3 L utility tariff [27]. This has been demonstrated as suitable for cost-utility analyses [28]. In both parts of the EQ-5D-3 L, higher values are associated with a better HRQL.

### Comorbidities

Participants were asked: “Has a physician ever diagnosed you with one of the following diseases?”. Thereby information on 33 pre-defined comorbidities was assessed. Comorbidities were added up to a comorbidity count. Such a count has been shown to perform well in quantifying comorbidity in COPD patients [29].

### Statistical analysis

For comparing characteristics between AATD patients and COPD control subjects, or between the two subgroups of AATD patients, t-tests for continuous, Chi^2^-tests for categorical variables, and Wilcoxon signed-rank tests for ordinal variables were applied.

The association of AATD and AT with direct and indirect costs was examined via generalised linear regression modelling (GLM). The covariates GOLD grade 1–4, age, sex, school education, smoking status, BMI and comorbidity count were added to the model. As cost data was highly right-skewed, which means that costs only assume values ≥0 and more individuals have costs below the mean than above, a gamma model with log-link was assumed. In the case of indirect costs only participants aged younger than 65 years were included. Participants with zero-costs were assigned €1 for regression modeling. Resulting cost estimates can be directly interpreted as factors. Adjusted means in direct and indirect costs were estimated as recycled predictions from 1000 bootstrap replications [30]. GLM with normal distribution was performed for HRQL scores. Therefore, estimates for HRQL represent additional units on the HRQL scale.

Statistical analyses were performed using SAS software (SAS Institute Inc., Cary, NC, USA, version 9.3). For significance testing, a *p*-value of 0.05 or less was required. Graphics were edited in R [31] using the package ggplot2 [32].

### Sensitivity analysis

To consider potential residual confounding due to the categorization of lung function in GOLD grades 1 to 4, GLM was also performed using the exact predicted percentage of Forced Expiratory Volume in 1 second (FEV_1_ %pred.) instead of GOLD grades [18].

### Ethics statement

The *COSYCONET* study was approved by the Ethics Committees of the local study centres. All participants gave their written informed consent.

## Results

### Characteristics of the study population

Data of 2049 COPD patients without AATD and 131 with AATD (106 with AT and 25 without AT) were included in the analysis. Their characteristics are given in Table 1.

**Table 1:** Characteristics of the study population

	Subgroups				*p*-value	
	A	B	B1	B2	A vs. B	B1 vs. B2
N	2049	131	106	25		
Age (years)	65.4 (8.2)	60.3 (10.0)	59.6 (9.9)	63.1 (10.2)	<.001^a^	.12^a^
% Age < 45 years	1.0	3.8	3.8	4.0	<.001^b^	.63 ^b^
% Age 45–55 years	9.1	29.0	30.19	24.0		
% Age 56–65 years	33.6	29.8	31.1	24.0		
% Age 66–75 years	44.1	31.3	30.2	36.0		
% Age > 75 years	12.1	6.1	4.72	12.0		
Gender						
% Males	61.1	56.5	62.3	32.0	.30^b^	.01^b^
COPD GOLD Grade					.01^b^	<.001^b^
% Grade 1 (FEV_1_% pred. ≥ 80%)	9.0	4.6	4.7	4.0		
% Grade 2 (50 ≤ FEV_1_% pred. < 80)	42.3	34.4	24.5	76.0		
% Grade 3 (30 ≤ FEV_1_% pred. < 50)	38.1	42.8	50.9	8.0		
% Grade 4 (FEV_1_% pred. < 30)	10.5	18.3	19.8	12.0		
Education					<.001^b^	.86^b^
% Basic school education	57.0	32.1	33.0	28.0		
% Secondary school education	26.0	43.5	42.5	48.0		
% Higher school education	17.0	24.4	24.5	24.0		
Smoking status					<.001^b^	.25^b^
% Never smokers	5.4	23.7	20.8	36.0		
% Former smokers	69.2	75.6	78.3	64.0		
% Smokers	25.4	0.7	0.9	0.0		
BMI (kg/m^2^)	26.8 (5.3)	24.7 (4.0)	24.6 (4.1)	24.8 (3.6)	<.001^a^	.85^a^
% Normal weight (18.5 ≤ BMI < 25)	35.7	52.7	52.8	52.0	<.001^b^	.64^b^
% Overweight (25 ≤ BMI < 30)	37.1	33.6	32.1	40.0		
% Obese (BMI ≥ 30)	23.7	9.9	10.4	8.0		
% Underweight (BMI < 18.5)	3.5	3.8	4.7	0.0		
Comorbidities						
Count	3.9 (2.6)	3.1 (2.5)	3.0 (2.5)	3.4 (2.3)	<.001^c^	.30^c^
% Liver cirrhosis	1.3	1.5	1.9	0.0	.68^b^	.49^b^
% Hepatitis	5.4	6.9	8.5	0.0	.48^b^	.13^b^

Data are mean (standard deviation) or percentage (%)

A = COPD patients without Alpha-1-antitrypsin deficiency (AATD), B = COPD patients with AATD, B1 = COPD patients with AATD and augmentation therapy (AT), B2 = COPD patients with AATD but without AT

^a^ *p*-value based on *t*-test, ^b^ *p*-value based on Chi^2^-test, ^c^Wilcoxon signed rank test

AATD patients were significantly younger (60.3 vs. 65.4 years, *p* < 0.001) and in higher GOLD grades than the COPD patients without AATD (*p* = 0.01), but showed a lower number of comorbidities (3.1 vs. 3.9, *p* < 0.001). Furthermore, AATD patients had a higher proportion of never-smokers (23.7% vs. 5.4%, *p* < 0.001) and a lower BMI (24.7 vs. 26.8 kg/m^2^, p < 0.001). Comparing AATD patients receiving AT with AATD patients not receiving AT, significant differences regarding the distributions of GOLD grades and gender were found (Table 1).

### Unadjusted healthcare utilisation, work absence and retirement

AATD patients, compared to COPD patients without AATD, had about twice as many outpatient visits, a lower percentage of hospitalizations (24% vs. 39%), a shorter duration of stay at inpatient services, and a lower number of prescribed medication (excluding AT) (Table 2). Also, AATD patients had less sick days and a lower proportion of premature retirement than COPD patients without AATD.

**Table 2:** Unadjusted healthcare utilization, costs and health-related quality of life

Healthcare utilization	Subgroups			
	A	B	B1	B2
	(*n* = 2049)	(*n* = 131)	(*n* = 106)	(*n* = 25)
Outpatient services (3 months)				
*General practitioner*				
% User	76	66	64	76
Number of visits	1.9 (2.3)	3.8 (4.8)	4.1 (5.0)	2.7 (3.7)
*Specialists*				
% User	94	96	96	96
Number of visits	4.4 (4.4)	8.9 (7.4)	9.6 (7.0)	5.9 (8.5)
*Ambulant hospital visits*				
%User	16	18	18	16
Number of visits	0.3 (1.5)	0.7 (3.0)	0.8 (3.3)	0.2 (0.4)
Inpatient services (12 months)				
% User	39	24	24	24
Number of visits	0.6 (1.1)	0.3 (0.8)	0.4 (0.9)	0.3 (0.5)
Duration of stay	5.8 (14.1)	2.3 (5.8)	2.2 (5.7)	2.7 (6.3)
Rehabilitation (12 months)				
*Ambulant*				
% User	2	4	3	8
Number of visits	0.5 (4.2)	0.6 (3.1)	0.5 (2.8)	1.16 (4.0)
*Stationary*				
% User	14	21	22	16
Duration of stay	3.3 (8.8)	5.2 (10.9)	5.4 (10.6)	5.0 (12.3)
Physiotherapist (12 months)				
%User	35	52	51	56
Number of visits	7.1 (18.5)	15.2 (23.6)	15.2 (24.1)	14.9 (22.1)
Medication (7 days)				
Number of prescribed medications (without AT)	6.1 (3.2)	4.7 (2.7)	4.7 (2.6)	4.4 (3.1)
Direct costs (12 months)				
Outpatient costs	938 (834)	2160 (1547)	2423 (1559)	1044 (857)
Inpatient costs	3441 (8302)	1341 (3394)	1285 (3337)	1579 (3687)
Rehabilitation	433 (1086)	674 (1327)	675 (1293)	666 (1487)
Physiotherapist	118 (307)	252 (393)	253 (400)	247 (368)
Medication costs	2533 (3327)	1961 (1076)	2056 (1080)	1558 (976)
Medication costs including AT	2533 (3327)	60427 (28718)	74311 (1080)	1558 (976)
Total direct costs	7446 (9836)	6374 (4535)	6675 (4202)	5096 (5655)
Total direct costs incl. AT	7446 (9836)	64840 (29467)	78930 (4202)	5096 (5655)
Indirect costs (participants < 65 years)	Subgroups			
	A	B	B1	B2
	(*n* = 897)	(*n* = 82)	(*n* = 69)	(*n* = 13)
Work absenteeism (12 months)				
% retired	47	35	37	23
% employed	38	55	54	62
% thereof with sick days (12 months)	73	67	59	100
Number of sick days	30.8 (50.9)	24.8 (46.7)	24.2 (50.1)	27.6 (28.0)
Indirect costs (12 months)				
Costs due to sick days (HC)	5452 (9015)	4395 (8278)	4287 (8882)	4895 (4965)
Costs due to sick days (FC)	2541 (2585)	2161 (2392)	1935 (2374)	3208 (2338)
Premature retirement (HC)	17508 (18543)	13129 (17859)	13989 (18123)	8568 (16280)
Total indirect costs (HC)	19514 (17615)	15541 (17231)	16288 (17595)	11580 (15155)
Total indirect costs (FC)	935 (1989)	1186 (2069)	1038 (1982)	1974 (2413)
Health-related Quality of Life	Subgroups			
	A	B	B1	B2
	(*n* = 2049)	(*n* = 131)	(*n* = 106)	(*n* = 25)
SGRQ	44.0 (20.0)	44.8 (17.2)	46.6 (16.4)	37.5 (20.2)
CAT	18.3 (7.4)	18.6 (6.7)	18.9 (6.6)	17.2 (7.3)
EQ-5D-3 L utility	81.5 (20.7)	83.2 (19.1)	83.0 (19.1)	83.9 (19.4)
EQ-5D VAS	56.2 (19.4)	56.4 (19.0)	54.4 (18.8)	63.6 (18.75)

Data are mean (standard deviation) or percentage (%)

A = COPD patients without Alpha-1-antitrypsin deficiency (AATD), B = COPD patients with AATD, B1 = COPD patients with augmentation therapy (AT), B2 = COPD patients with AATD but without AT, HC = human capital approach, FC = friction costs approach

Number of visits is including all participants, also those who have 0 visits

Total direct and indirect costs differ from the sum score calculated from the single categories due to differences in n between the single categories. EQ-5D-3 L utility is displayed after multiplication with 100

Total direct costs do not include AT costs of approximately €72,000

AATD patients receiving AT compared to AATD patients not receiving AT visited a general practitioner or specialist about two times more often and had a higher rate of premature retirement (37% vs. 23%). It has to be noted that the number of outpatient visits of AT receiving AATD patients did most likely include visits for AT application as information on the purpose of visits was missing.

### Association of AATD and AT with direct and indirect costs

All cost estimates considered in the following analyses exclude the costs of AT therapy; thus estimated mean annual costs of approximately €72,000 [21] have to be added in patients receiving AT.

Table 3 shows the major results of regression models for costs. Adjusted for possible confounders, there were no significant associations of AATD (with or without AT) with total direct costs. However, AATD patients with AT showed significantly higher annual outpatient costs (factor 2.7, *p* < 0.001) compared to patients without AATD. Conversely, AT-receiving patients had lower inpatient costs (−35%, *p* = 0.02). Medication costs, excluding AT costs, were decreased by 10% (*p* = 0.11) and 28% (*p* = 0.01) in AATD patients with and without AT, respectively. As stated before, these results were not adjusted for the reason of consultation. Therefore, the number of outpatient visits in AATD patients with AT may include a number of visits that were exclusively due to AT administration. There were no significant associations of AATD with indirect cost neither for the human capital nor the friction cost approach. Complete regression results are shown in Additional file 2.

**Table 3:** Results of regression models: Association of AATD and AT with annual direct and indirect costs

		Direct costs					Indirect costs	
Costs		Total direct costs	Outpatient costs	Inpatient costs	Medication costs	Other costs	HC	FC
Group	A	Ref.	Ref.	Ref.	Ref.	Ref.	Ref.	Ref.
	B1	0.95 (0.80 – 1.12)	**2.73** (2.35 – 3.17)	**0.65** (0.45 – 0.94)	0.90 (0.79 – 1.03)	1.14 (0.81 – 1.61)	1.05 (0.79 – 1.39)	0.94 (0.66 – 1.35)
	B2	0.80 (0.57 – 1.13)	1.13 (0.84 – 1.51)	0.79 (0.39 – 1.63)	**0.72** (0.56 – 0.93)	1.23 (0.65 – 2.34)	0.67 (0.38 – 1.18)	1.18 (0.67 – 2.08)

A = COPD patients without Alpha-1-antitrypsin deficiency (AATD), B1 = COPD patients with AATD and augmentation therapy (AT), B2 = COPD patients with AATD but without AT

Indirect costs only include patients < 65 years of age

Significant estimates on a level of *p* < .05 are printed bold

Other costs include Physiotherapist and Rehabilitation costs

*HC* Human capital approach, *FC* Friction costs approach

Total direct costs and medication costs do not include AT costs of approximately €72,000

**Fig. 2:**
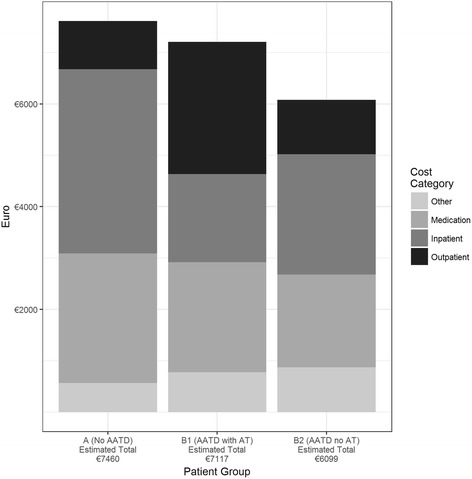
Displays the four direct cost categories in relation to the total amount of direct costs. Patients with Alpha-1-antitrypsin deficiency (AATD) and augmentation therapy (AT) have lower inpatient but also higher outpatient costs, compared to the two other subgroups. Therefore, no significant differences could be detected between the three groups. However, AATD patients - especially non AT receiving AATD patients - tended to have lower direct costs

Adjusted mean annual direct and indirect costs are shown in Table 4. Total annual direct costs due to healthcare, were highest in COPD patients without AATD (€7460 [€7026 – €7924]) and lowest in patients with AATD but without AT €6099 ([€4288 – €8256]). AATD patients with AT had mean annual direct cost of €7117 ([€6029 – €8392]). The categories of direct costs, in relation to the total amount of direct costs in the three subsamples, are illustrated in Fig. 2. Inpatient costs contributed most to the total healthcare costs in COPD patients without AATD (€3586 [€3201 – €4010]) and AATD patients without AT (€2338 [€561 – €5288]). Most cost driving category in AATD patients with AT was outpatient costs (€2570 [€2256 – €2866]).

**Table 4:** Adjusted annual direct and indirect costs

Adjusted mean annual costs (€)					
Direct costs	Total	Outpatient costs	Inpatient costs	Medication costs	Other costs
% zero costs	0%	0%	62%	0%	56%
Group					
A	7460 [7026 – 7924]	937 [902 – 973]	3586 [3201 – 4010]	2522 [2381 – 2663]	566 [513 – 628]
B1	7117 [6029 – 8392]	**2570 [2256 – 2866]**	**1715 [740 – 3076]**	**2144 [1903 – 2409]**	777 [529 – 1049]
B2	6099 [4288 – 8256]	1061 [763 – 1414]	2338 [561 – 5288]	**1805 [1388 – 2261]**	876 [319 – 1659]
Adjusted mean annual costs (€)					
Indirect costs		Human capital approach		Friction costs approach	
% zero costs		26%		72%	
Group					
A		19,583 [18,451 – 20,803]		981 [839 – 1138]	
B1		18,813 [13,632 – 24,716]		1276 [608 – 2181]	
B2		16,171 [6194 – 28,316]		1866 [633 – 3764]	

A = COPD patients without Alpha-1-antitrypsin deficiency (AATD), B1 = COPD patients with AATD and augmentation therapy (AT), B2 = COPD patients with AATD but without AT

Significant estimates on a level of *p* < 0.05 are printed bold. Indirect costs only include participants < 65 years of age

Totals direct costs and medication costs do not include AT costs of approximately €72,000

### Comparison of HRQL in the three subgroups

**Fig. 3:**
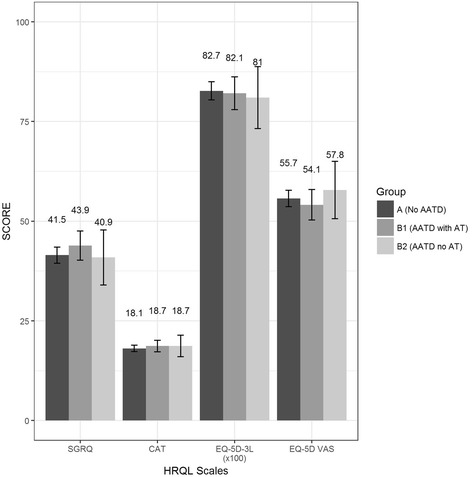
Displays the four HRQL scales in comparison between the three subgroups. No differences could be detected. AATD = Alpha-1-antitrypsin deficiency, AAT = augmentation therapy

There were no significant differences regarding HRQL between the groups. In particular, having AATD with or without AT was not associated with differences in CAT, SGRQ, EQ-5D-3 L utility or EQ-5D VAS compared to patients without AATD. Results from regression analyses are shown in Table 5; detailed regression results can be found in Additional file 3. Adjusted mean HRQL scores are provided in Fig. 3.

**Table 5:** Results of regression models: Regression coefficients of AATD patients with and without AT

Regression coefficients					
HRQL		SGRQ	CAT	EQ-5D-3 L utility	EQ-5D VAS
Group	A	Ref.	Ref.	Ref.	Ref.
	B1	2.68 (−0.82 – 6.19)	0.66 (−0.73 – 2.04)	−0.55 (−4.45 – 3.35)	−1.58 (−5.23 – 2.07)
	B2	−0.64 (−7.53 – 6.24)	0.60 (−2.09 – 3.31)	−1.67 (−9.34 – 6.00)	2.14 (−5.01 – 9.30)

Group A = COPD patients without Alpha-1-antitrypsin deficiency (AATD), Group B1 = COPD patients with AATD and augmentation therapy (AT), B2 = COPD patients with AATD but without augmentation therapy

EQ-5D-3 L utility was multiplied by 100

Significant estimates on a level of *p* < 0.05 are printed bold

### Result of sensitivity analysis

Using FEV_1_ in terms of the exact %predicted instead of GOLD grades 1 to 4 for adjustment in regression models did not affect the association between patient groups and costs or HRQL. Thus there is no indication of residual confounding by using the GOLD grades 1 to 4 instead of the continuous variable FEV_1_%pred. Details are shown in Additional files 4 and 5.

## Discussion

This study compared costs and HRQL between COPD patients with AATD (with or without AT) and COPD patients without this deficiency. Costs of AT therapy (about €72,000 per year [21]) were excluded in order to identify the healthcare costs as a function of the patient group. AATD patients, both with and without AT, tended to have lower total costs compared to COPD patients without AATD, but these differences did not reach statistical significance. Accordingly, most of the single cost categories also did not show significant differences. However, adjusted mean medication costs were significantly lower in both AATD patient groups compared to COPD patients without AATD. Outpatient costs were considerably increased in AATD patients with AT, while inpatient costs were lower. There were no differences found between the groups regarding indirect costs or HRQL.

A recent study by Greulich et al. [33] that examined German claims data of about 2.8 million insurants over a time period of four years conducted an age- and gender-matched (1:10) comparison between 5,900 COPD patients without AATD and 590 COPD patients with AATD. AATD patients had more outpatient consultations and a higher hospitalization rate compared to COPD patients without AATD [33]. These results were not adjusted for disease severity or stratified by AT which might explain the, compared to our results, opposite finding regarding the hospitalization rate of AATD patients. With respect to the higher frequency of outpatient service consultation in AATD patients, Greulich et al., like us, point out that this is at least in part explainable by AT application [33].

Another study by Zacherle et al. using US claims data also found significantly more inpatient visits in AATD patients compared to COPD patients without AATD (58% vs. 19.5%) leading to an increase by $US27,674 in total healthcare costs per patient [34]. However, this study included the AT costs of AT-receiving AATD patients (13% in the sample) and was not adjusted for differences in COPD severity. In another US survey Mullins et al. performed a cost analysis on AATD patients in 2003 and identified mean costs per year for hospitalization, outpatient services and prescription of drugs (other than AT) of $US4,497, $US2,299, and $US6,456, respectively [35]. These costs are higher than the costs estimated for the COSYCONET dataset. The differences could be due to a lack of adjustment for case severity and to national cost differences. In COSYCONET, indirect costs did not show significant differences between COPD patients with and without AATD. However, Zacherle et al. found COPD patients without AATD to receive disability benefits twice as often as AATD patients (40.1% vs. 20.3%, *p* < 0.001) [34]. To the knowledge of the authors, the present study is the first one comparing indirect costs in COPD patients with and without AATD in a comprehensive manner.

After stratification of AATD patients for AT, some differences appeared regarding single direct cost factors. Patients receiving AT showed considerably increased outpatient but decreased inpatient costs compared to COPD patients and AATD patients not receiving AT. Other studies relevant for inpatient or outpatient costs examined exacerbation rates or FEV_1_ and showed inconsistent or not significant results [15]. The increase of outpatient costs in patients receiving AT in our study is at least partially explainable by the AT itself which is usually administered by a weekly intravenous infusion in the outpatient setting. As we did not have information on the reason for individual physician visits, these costs could not be specified in our analysis. Conversely, the decreased inpatient and medication costs might be a beneficial side-effect of the more frequent outpatient visits associated with the AT. The more frequent visits at outpatient services might lead to a better adherence to self-management plans and compliance to medication plans. Furthermore, they might facilitate the early detection of changes in needs and clinical picture of the patients and therefore prevent hospitalisation. This effect may also, at least partially, explain the overall although not significant lower costs in AATD patients.

Our finding that HRQL did not differ between COPD patients with and without AATD is in line with former research by Manca et al. who did not find HRQL differences between AATD patients and COPD patients without AATD, regarding CAT, EQ-5D-3 L and EQ-5D VAS [36]. HRQL also did not differ between AATD patients with and without AT. Similar results were found in a randomized, placebo-controlled trial by Chapman et al. [37] in which SGRQ values did not differ significantly (44.3 vs. 42.4, *p* = 0.91) between AATD patients with and without AT. The results presented in the review by Gøtzsche et al. also suggest no clinically relevant differences in HRQL (41).

When considering the cost reductions due to less usage of inpatient services and prescribed medication in AT-receiving AATD patients, it has to be kept in mind that these patients cause very high total annual costs of about €72,000 due to augmentation therapy. Our study shows that this amounts for about 91% of the total direct costs in AATD patients receiving AT. In our opinion, the finding that HRQL did not differ between AATD patients with AT and COPD patients without AATD does not allow conclusions about the effect of AT on HRQL. In particular AATD patients not receiving AT could not serve as a control group for AT-receiving AATD patients, as we did not have access to the detailed information that had led to the prescription of AT. The AATD patients without AT were older and had less severe COPD compared to AATD patients with AT. This could be partly explained by therapeutic guidelines, which indicate to prescribe AT only in severe cases with minimal smoking history [38]. Nevertheless, our results underline how important information on treatment effects of AT on HRQL of AATD patients is both medically and economically. Thus clarification of the (cost-) effectiveness of AT should have high priority in AATD research.

There have been several efforts to estimate the cost effectiveness of AT, however with inconsistent results [39, 40]. From the perspectives of both health economics and morbidity profiling it is of interest whether the costs of standard COPD therapy depend on the presence of AATD and the use of AT. We provide such data regarding single direct and indirect cost factors, and the results can be used as more precise estimators of yearly costs in the three patient groups.

Recently there has been new supporting evidence for the effectiveness of AT in AATD therapy by a randomized clinical trial [41]. However, our finding of indifferent costs also raises the question how a potentially intensified but standard COPD therapy compares with additional AT and which long-term health and cost benefits might be associated with these options. Due to AATD patients often being excluded from pharmacologic trials on COPD therapy, there is actually a lack of evidence on the impact of standard therapy on this patient group.

The strength of the study is based on a sample size that enabled us to include sufficient numbers of patients in all three patient groups, with respect to the rarity of AATD. Furthermore, the COSYCONET COPD cohort acquired detailed information to analyse direct and indirect costs, as well as generic and disease-specific HRQL. There are, however, also limitations. The analysis of healthcare utilisation relied on self-reported questionnaires, with the risk of recall bias. However, there were no hints for differential biases between groups. Some healthcare services such as nursing, medical aids and appliances were not considered in the cost calculations. Finally, in this cross-sectional study only statistical associations could be explored. Despite the limitations it is reasonable to assume that the estimates derived from the baseline visit of COSYCONET give a realistic picture of direct and indirect costs as well as of the HRQL in COPD with and without AATD and AT.

## Conclusion

If AT costs were excluded, AATD patients tended to show lower, but not significant, overall costs and similar HRQL compared to COPD patients without AATD. AT was not associated with lower costs or higher HRQL from a cross-sectional perspective.

## Additional files

Additional file 1: ^a^German unit costs according to Bock et al. [19]. ^b^Indirect costs only for subjects of employable age < 65 years. ^c^Work absence only for full-time and regular part-time employees. (DOC 40 kb)

Additional file 2: Indirect costs only include participants < 65 years of age. Significant estimates on a level of *p* < .05 are printed bold. A = COPD patients without AATD, B1 = COPD patients with Alpha-1-antitrypsin deficiency (AATD) and augmentation therapy (AT), B2 = COPD patients with AATD but without AT. Other costs include physiotherapist and rehabilitation costs. (DOC 68 kb)

Additional file 3: Significant estimates on a level of *p* < .05 are printed bold. A = COPD patients without Alpha-1-antitrypsin deficiency (AATD), B1 = COPD patients with AATD and augmentation therapy (AT), B2 = COPD patients with AATD but without AT. EQ-5D-3 L values are given after multiplication by 100. (DOC 47 kb)

Additional file 4: Indirect costs only include participants < 65 years of age. Significant estimates on a level of *p* < .05 are printed bold. A = COPD patients without Alpha-1-antitrypsin deficiency (AATD), B1 = COPD patients with AATD and augmentation therapy (AT), B2 = COPD patients with AATD but without AT. Other costs include physiotherapist and rehabilitation costs. There was no relevant impact of the change in the model on the estimates of the different groups. (DOC 28 kb)

Additional file 5: Significant estimates on a level of p < .05 are printed bold. A = COPD patients without Alpha-1-antitrypsin deficiency (AATD), B1 = COPD patients with AATD and augmentation therapy (AT), B2 = COPD patients with AATD but without AT. (DOC 29 kb)

## Abbreviations

AATD: Alpha-1-Antitrypsin Deficiency
AT: Augmentation Therapy
BMI: Body Mass Index
CAT: COPD Assessment Test
COPD: Chronic Obstructive Pulmonary Disease
COSYCONET: German COPD and Systemic Consequences – Comorbidities Network
EQ-5D-3 L: EuroQol 5 dimensions, 3 level version
FEV_1_ %pred.: Forced Expiratory Volume in 1 second in percentage of predicted
GLI: Global Lung Initiative
GLM: Generalized linear regression model
GOLD: Global Initiative for Chronic Obstructive Pulmonary Disease
HRQL: Health-related quality of life
SGRQ: Saint-George’s-Respiratory-Questionnaire
VAS: Visual Analogue Scale.
