# Supplementary material for: Costs and health-related quality of life in Alpha-1-Antitrypsin Deficient COPD patients
Source: Respir Res. 2017 Apr 17;18:60. doi: 10.1186/s12931-017-0543-8 (PMC5392996; doi:10.1186/s12931-017-0543-8)
Supplement: Supplementary file 1 — aGerman unit costs according to Bock et al. [19]. bIndirect costs only for subjects of employable age < 65 years. cWork absence only for full-time and regular part-time employees. (DOC 40 kb) [file 12931_2017_543_MOESM1_ESM.doc]

**Additional file 1**

|  | Unit costs (€) per day/visit |
| --- | --- |
| **Direct costs categories**^a^ |  |
| General practitioner or GP for internal medicine | 20.22 |
| Specialists |  |
| Specialist for internal medicine (incl. pneumologist) | 64.65 |
| Gynecologist | 30.53 |
| Ophthalmologist | 35.09 |
| Orthopedist | 25.27 |
| Otorhinolaryngologist | 27.55 |
| Surgeon | 44.11 |
| Dermatologist | 19.10 |
| Radiologist | 43.97 |
| Urologist | 24.97 |
| Neurologist/psychiatrist / Psychotherapist | 45.58 |
| Specialist in occupational medicine | 20.22 |
| Other physician (incl. ambulant hospital visits) | 43.97 |
| Rehabilitation |  |
| Ambulant visit | 48.29 |
| Inpatient rehabilitation | 122.09 |
| Hospital treatment |  |
| Ambulant visit | 43.97 |
| Inpatient hospital | 589.32 |
| Physiotherapist | 16.62 |
| Prescribed pharmaceuticals | AOK Institute |
| **Indirect cost categories**^b^ |  |
| Work absence^c^ | 177.21 |
| Premature retirement | 37,126 (per year) |
