# Supplementary material for: Costs and health-related quality of life in Alpha-1-Antitrypsin Deficient COPD patients
Source: Respir Res. 2017 Apr 17;18:60. doi: 10.1186/s12931-017-0543-8 (PMC5392996; doi:10.1186/s12931-017-0543-8)
Supplement: Supplementary file 2 — Indirect costs only include participants < 65 years of age. Significant estimates on a level of p < .05 are printed bold. A = COPD patients without AATD, B1 = COPD patients with Alpha-1-antitrypsin deficiency (AATD) and augmentation therapy (AT), B2 = COPD patients with AATD but without AT. Other costs include physiotherapist and rehabilitation costs. (DOC 68 kb) [file 12931_2017_543_MOESM2_ESM.doc]

**Additional file 2**

|  | Direct Costs | | | | | Indirect costs | |
| --- | --- | --- | --- | --- | --- | --- | --- |
|  | Total direct costs | Outpatient costs | Inpatient costs | Medication costs | Other costs | HC | FC |
| Group |  |  |  |  |  |  |  |
| A | ref. | ref. | ref. | ref. | ref. | ref. | ref. |
| B1 | 0.95 (0.80 – 1.12) | 2.73 (2.35 – 3.17) | 0.65 (0.45 – 0.94) | 0.90 (0.79 – 1.03) | 1.14 (0.81 – 1.61) | 1.05 (0.79 – 1.39) | 0.94 (0.66 – 1.35) |
| B2 | 0.80 (0.57 – 1.13) | 1.13 (0.84 – 1.51) | 0.79 (0.39 – 1.63) | 0.72 (0.56 – 0.93) | 1.23 (0.65 – 2.34) | 0.67 (0.38 – 1.18) | 1.18 (0.67 – 2.08) |
| COPD GOLD grade |  |  |  |  |  |  |  |
| Grade 1 | ref. | ref. | ref. | ref. | ref. | ref. | ref. |
| Grade 2 | **1.19** (1.05 – 1.36) | 1.00 (0.89 – 1.12) | 1.18 (0.92 – 1.52) | 1.10 (0.99 – 1.21) | 1.16 (0.86 – 1.58) | 1.08 (0.82 -1.41) | 1.03 (0.75 – 1.43) |
| Grade 3 | **1.75** (1.53 – 2.00) | 1.03 (0.92 – 1.16) | **1.72** (1.34 – 2.22) | **1.35** (1.22 – 1.49) | **1.48** (1.09 – 2.01) | **1.39** (1.06 – 1.83) | 1.26 (0.90 – 1.77) |
| Grade 4 | **2.33** (1.98 – 2.74) | 1.02 (0.89 – 1.18) | **2.16** (1.63 – 2.87) | **1.50** (1.33 – 1.70) | **1.76** (1.21 – 2.55) | **1.70** (1.25 – 2.31) | 1.07 (0.68 – 1.68) |
| Age |  |  |  |  |  |  |  |
| < 45 years | ref. | ref. | ref. | ref. | ref. | ref. | ref. |
| 45 - 55 years | 1.20 (0.85 – 1.69) | 0.95 (0.71 – 1.29) | 1.63 (0.78 – 3.38) | 1.16 (0.89 – 1.49) | 0.93 (0.48 – 1.79) | 1.23 (0.78 – 1.94) | 1.00 (0.64 – 1.56) |
| 56 - 65 years | 1.15 (0.83 – 1.60) | 0.93 (0.70 – 1.25) | 1.46 (0.71 – 2.98) | 1.27 (0.99 – 1.62) | 0.88 (0.47 – 1.65) | **1.88** (1.22 – 2.91) | 1.05 (0.68 – 1.61) |
| 66 – 75 years | 1.12 (0.80 – 1.56) | 0.90 (0.68 – 1.21) | 1.55 (0.76 – 3.16) | 1.18 (0.92 – 1.51) | 0.76 (0.41 – 1.43) | - | - |
| > 75 years | 1.05 (0.74 – 1.48) | 0.92 (0.68 – 1.24) | 1.54 (0.74 – 3.21) | 1.16 (0.90 – 1.50) | 0.63 (0.32 – 1.21) | - | - |
| Sex |  |  |  |  |  |  |  |
| Female | ref. | ref. | ref. | ref. | ref. | ref. | ref. |
| Male | 1.05 (0.98 – 1.14) | **0.92** (0.86 - 0.98) | 1.01 (0.89 – 1.16) | 0.99 (0.93 – 1.04) | **1.23** (1.04 – 1.45) | 0.98 (0.85 – 1.13) | 1.10 (0.90 – 1.34) |
| Education |  |  |  |  |  |  |  |
| Basic | ref. | ref. | ref. | ref. | ref. | ref. | ref. |
| Secondary | 1.08 (0.99 – 1.18) | 0.97 (0.90 – 1.04) | 1.12 (0.97 – 1.29) | 1.02 (0.96 – 1.09) | 1.04 (0.86 – 1.26) | **0.85** (0.72 – 0.99) | 0.97 (0.78 – 1.21) |
| Higher | 0.99 (0.90 – 1.1) | 1.00 (0.92 – 1.04) | 1.00 (0.84 – 1.19) | 0.98 (0.91 – 1.06) | 0.92 (0.74 – 1.14) | **0.76** (0.62 – 0.92) | 0.85 (0.65 – 1.10) |
| Smoking status |  |  |  |  |  |  |  |
| Never smoker | ref. | ref. | ref. | ref. | ref. | ref. | ref. |
| Former smoker | **1.18** (1.01 – 1.37) | 1.10 (0.97 – 1.25) | 0.79 (0.58 – 1.09) | **1.14** (1.01 – 1.27) | 1.11 (0.80 – 1.55) | 1.06 (0.77 – 1.48) | 1.03 (0.66 – 1.61) |
| Smoker | 0.94 (0.79 – 1.11) | 1.05 (0.91 – 1.21) | 0.82 (0.58 – 1.16) | 0.92 (0.82 – 1.05) | 1.03 (0.71 – 1.50) | 1.03 (0.73 – 1.46) | 1.15 (0.73 – 1.82) |
| BMI |  |  |  |  |  |  |  |
| Normal weight | ref. | ref. | ref. | ref. | ref. | ref. | ref. |
| Overweight | **1.14** (1.05 – 1.24) | 1.02 (0.95 – 1.10) | 1.13 (0.98 – 1.31) | 1.04 (0.98 – 1.11) | 1.16 (.96 – 1.40) | 0.97 (0.83 – 1.15) | 1.03 (0.82 – 1.29) |
| Obese | **1.12** (1.01 – 1.24) | 1.04 (0.95 – 1.13) | 1.14 (0.97 – 1.35) | **1.09** (1.01 – 1.17) | 1.01 (.81 – 1.26) | 1.02 (0.85 – 1.22) | 1.13(0.88 – 1.47) |
| Underweight | 1.07 (0.87 – 1.30) | 0.91 (0.77 – 1.08) | 1.29 (0.92 – 1.81) | 0.93 (0.80 – 1.08) | 1.25 (0.81 – 1.93) | 0.99 (0.70 – 1.40) | 0.77 (0.45 – 1.33) |
| Comorbidities | **1.11** (1.10 – 1.13) | **1.08** (1.06 – 1.09) | **1.05** (1.03 – 1.08) | **1.09** (1.08 – 1.10 | **1.91** (1.82 – 2.01) | **1.04** (1.01 – 1.07) | **1.06** (1.01 – 1.10 |
