# Supplementary material for: Costs and health-related quality of life in Alpha-1-Antitrypsin Deficient COPD patients
Source: Respir Res. 2017 Apr 17;18:60. doi: 10.1186/s12931-017-0543-8 (PMC5392996; doi:10.1186/s12931-017-0543-8)
Supplement: Supplementary file 3 — Significant estimates on a level of p < .05 are printed bold. A = COPD patients without Alpha-1-antitrypsin deficiency (AATD), B1 = COPD patients with AATD and augmentation therapy (AT), B2 = COPD patients with AATD but without AT. EQ-5D-3 L values are given after multiplication by 100. (DOC 47 kb) [file 12931_2017_543_MOESM3_ESM.doc]

**Additional file 3**

|  | Health-related Quality of Life | | | |
| --- | --- | --- | --- | --- |
|  | SGRQ | CAT | EQ-5D-3L | EQ-5D VAS |
| Group |  |  |  |  |
| A | ref. | ref. | ref. | ref. |
| B1 | 2.68 (-0.82 – 6.19) | 0.66 (-0.73 – 2.04) | -0.55 (-4.45 – 3.35) | -1.58 (-5.23 – 2.07) |
| B2 | -0.64 (-7.53 – 6.24) | 0.60 (-2.09 – 3.31) | -1.67 (-9.34 – 6.00) | 2.14 (-5.01 – 9.30) |
| COPD GOLD grade |  |  |  |  |
| Grade 1 | ref. | ref. | ref. | ref. |
| Grade 2 | **10.88** | **2.53** | -1.05 | **-6.04** |
| Grade 3 | **21.22** | **5.14** | **-5.04** | **-14.96** |
| Grade 4 | **32.55** | **8.22** | **-13.93** | **-22.75** |
| Age |  |  |  |  |
| < 45 years | ref. | ref. | ref. | ref. |
| 45 - 55 years | **7.99** | 1.33 | -2.51 | -1.65 |
| 56 - 65 years | **8.32** | 0.91 | -2.82 | -2.10 |
| 66 – 75 years | **7.99** | -0.17 | -1.14 | -1.06 |
| > 75 years | **8.84** | -0.61 | -2.78 | **-2.84** |
| Sex |  |  |  |  |
| Female | ref. | ref. | ref. | ref. |
| Male | -0.44 | 0.01 | 0.70 | -1.82 |
| Education |  |  |  |  |
| Basic | ref. | ref. | ref. | ref. |
| Secondary | **-3.68** | **-1.01** | **2.08** | **3.36** |
| Higher | **-4.46** | **-1.67** | **4.91** | **2.85** |
| Smoking status |  |  |  |  |
| Never smoker | ref. | ref. | ref. | ref. |
| Former smoker | 1.87 | 0.45 | -2.34 | 0.43 |
| Smoker | 1.90 | 1.03 | -2.27 | -0.07 |
| BMI |  |  |  |  |
| Normal weight | ref. | ref. | ref. | ref. |
| Overweight | 1.51 | 0.13 | **-2.34** | -1.51 |
| Obese | **5.95** | **1.46** | **-5.13** | -4.49 |
| Underweight | 1.25 | 0.68 | -0.89 | -5.67 |
| Comorbidities | **1.77** | **0.65** | **-2.31** | **-1.34** |
