# Supplementary material for: Costs and health-related quality of life in Alpha-1-Antitrypsin Deficient COPD patients
Source: Respir Res. 2017 Apr 17;18:60. doi: 10.1186/s12931-017-0543-8 (PMC5392996; doi:10.1186/s12931-017-0543-8)
Supplement: Supplementary file 4 — Indirect costs only include participants < 65 years of age. Significant estimates on a level of p < .05 are printed bold. A = COPD patients without Alpha-1-antitrypsin deficiency (AATD), B1 = COPD patients with AATD and augmentation therapy (AT), B2 = COPD patients with AATD but without AT. Other costs include physiotherapist and rehabilitation costs. There was no relevant impact of the change in the model on the estimates of the different groups. (DOC 28 kb) [file 12931_2017_543_MOESM4_ESM.doc]

**Additional file 4**

| Covariate |  | Direct Costs | | | | | Indirect costs | |
| --- | --- | --- | --- | --- | --- | --- | --- | --- |
|  |  | Total direct costs | Outpatient costs | Inpatient costs | Medication costs | Other costs | HC | FC |
| Group | A | ref. | ref. | ref. | ref. | ref. | ref. | ref. |
|  | B1 | 0.93 (0.79 – 1.11) | **2.72** (2.35 – 3.16) | **0.66** (0.45 - 0.95) | 0.89 (0.78 – 1.01) | 1.15 (0.82 – 1.62) | 1.05 (0.79 – 1.39) | 0.97 (0.68 – 1.39) |
|  | B2 | 0.79 (0.56 – 1.10) | 1.12 (0.83 – 1.51) | 0.77 (0.38 – 1.58) | **0.70** (0.54 - 0.91) | 1.26 (0.66 – 2.38) | 0.63 (0.36 – 1.10) | 1.10 (0.62 – 1.94) |
| FEV_1_ % predicted |  | **0.99** (0.98 – 0.99) | 1.00 (1.00 – 1.00) | **0.99** (0.98 - 0.99) | **0.99** (0.99 – 0.99) | **0.99** (0.99 – 1.00) | 0.99 (0.98 – 1.00) | 1.00 (0.99 – 1.00) |
