# Supplementary material for: Costs and health-related quality of life in Alpha-1-Antitrypsin Deficient COPD patients
Source: Respir Res. 2017 Apr 17;18:60. doi: 10.1186/s12931-017-0543-8 (PMC5392996; doi:10.1186/s12931-017-0543-8)
Supplement: Supplementary file 5 — Significant estimates on a level of p < .05 are printed bold. A = COPD patients without Alpha-1-antitrypsin deficiency (AATD), B1 = COPD patients with AATD and augmentation therapy (AT), B2 = COPD patients with AATD but without AT. (DOC 29 kb) [file 12931_2017_543_MOESM5_ESM.doc]

**Additional file 5**

| Covariate |  | HRQL | | | |
| --- | --- | --- | --- | --- | --- |
|  |  | SGRQ | CAT | EQ-5D-3L | EQ-5D VAS |
| Group | A | ref. | ref. | ref. | ref. |
|  | B1 | 2.38 (-1.08 – 5.85) | 0.56 (-0.81 – 1.94) | -0.48 (-4.40 – 3.44) | -1.47 (-5.11 – 2.17) |
|  | B2 | -0.55 (-7.35 – 6.25) | 0.61 (-2.07 – 3.30) | -1.48 (-9.17 – 6.22) | 2.56 (-4.56 – 9.68) |
| FEV_1_%predicted |  | **1.78** (1.50 - 2.07) | **0.66** (0.54 - 0.77) | **-2.30** (-2.62 – 1.98) | **-1.35** (-1.65 – -1.06) |
